# A Pilot Microbiota Study in Parkinson’s Disease Patients versus Control Subjects, and Effects of FTY720 and FTY720-Mitoxy Therapies in Parkinsonian and Multiple System Atrophy Mouse Models

**DOI:** 10.3233/JPD-191693

**Published:** 2020-01-13

**Authors:** Guadalupe Vidal-Martinez, Brandon Chin, Cynthia Camarillo, Gloria V. Herrera, Barbara Yang, Irene Sarosiek, Ruth G. Perez

**Affiliations:** a Texas Tech University Health Sciences Center El Paso, Center of Emphasis in Neurosciences, Department of Molecular and Translational Medicine, Graduate School of Biomedical Sciences, El Paso, TX, USA; b Department of Internal Medicine, Division of Gastroenterology, Paul L Foster School of Medicine, El Paso, TX, USA

**Keywords:** Microbiota, Parkinson’s disease, multiple system atrophy, FTY720, FTY720-Mitoxy, transgenic mouse models

## Abstract

**Background:**

Parkinson’s disease (PD) and multiple system atrophy (MSA) patients often suffer from gastrointestinal (GI) dysfunction and GI dysbiosis (microbial imbalance). GI dysfunction also occurs in mouse models of PD and MSA.

**Objectives:**

To assess gut dysfunction and dysbiosis in PD subjects as compared to controls, identify potential shared microbial taxa in humans and mouse models of PD and MSA, and to assess the effects of potential therapies on mouse GI microbiota.

**Methods:**

In this human pilot study, GI function was assessed by fecal consistency/frequency measured using the Bristol Stool Form Scale and GI transit time assessed using Sitzmarks pills and abdominal radiology. Human and mouse microbiota were analyzed by extracting fecal genomic DNA followed by 16S rRNA sequencing.

**Results:**

In our PD patients genera *Akkermansia* significantly increased while a trend toward increased *Bifidobacterium* and decreased *Prevotella* was observed. Families Bacteroidaceae and Lachnospiraceae and genera *Prevotella* and *Bacteroides* were detected in both humans and PD mice, suggesting potential shared biomarkers. In mice treated with the approved multiple sclerosis drug, FTY720, or with our FTY720-Mitoxy-derivative, we saw that FTY720 had little effect while FTY720-Mitoxy increased beneficial *Ruminococcus* and decreased Rickenellaceae family.

**Conclusion:**

*Akkermansia* and Prevotellaceae data reported by others were replicated in our human pilot study suggesting the use of those taxa as potential biomarkers for PD diagnosis. The effect of FTY720-Mitoxy on taxa Rikenellaceae and *Ruminococcus* and the relevance of S24-7 await further evaluation. It also remains to be determined if mouse microbiota have predictive power for human subjects.

## INTRODUCTION

The gut microbiome plays an important role in regulating metabolism [1–3], immunity [4], and neurological function [5–7]. In addition to antibiotics, certain non-antibiotic drugs and/or common medications can alter gut microbial growth or elicit changes that may induce antibiotic resistance [8]. Gut microbial imbalances are also common in chronic diseases like diabetes [9, 10], inflammatory bowel disease [11], and obesity [12], as well as in neurodegenerative disorders like Parkinson’s disease (PD) [13–15] and multiple system atrophy (MSA) [16, 17]. Gut microbiome changes in PD and MSA often parallel associated gastrointestinal (GI) hyperpermeability, constipation, and dysmotility. Interestingly, such GI changes can begin years before gait/motor symptoms, suggesting that specific microbial signatures may yield predictive biomarkers for early PD or MSA diagnosis [18].

In order to assess the effect of potential therapeutics for treating PD and MSA pathology we obtained relevant mouse models. To model PD we used a parkinsonian mouse that expresses A53T mutant human alpha-synuclein (a-Syn) in neurons [19], referred to here as A53T transgenic (Tg) mice and an MSA mouse model that expresses human aSyn in oligodendrocytes under the 2, ^′^ 3^′^-cyclic nucleotide 3^′^-phosphodiesterase (CNP) promoter [20], referred to here as CNP-aSyn transgenic (Tg) mice. The A53T and the CNP-aSyn Tg mice mimic some of the neuropathology found in patients with PD and MSA, including age-onset motor and GI dysfunction [21, 22].

Other studies have suggested that GI dysfunction and dysbiosis are strongly related and that the intestine may be a major source leading to neuroinflammation [23, 24]. In PD patients and in parkinsonian animal models, aggregated a-Syn is found in neurons of the enteric nervous system as we have previously reported for A53T Tg mice [21, 22]. A53T and CNP-aSyn Tg mice are thus anticipated to have dysbiosis as they suffer GI dysfunction and/or a-Syn aggregation in the enteric nervous system in contrast to their wild type (WT) littermates that have no pathology or GI dysfunction and thus are expected to have normal gut microbiota.

Previously we and others have studied protection by FTY720, an approved oral immunosuppressive drug for multiple sclerosis, that when phosphorylated acts on sphingosine-1-phosphate receptors (S1P-Rs) [25]. FTY720 causes immunosuppression but also enhances expression of brain derived trophic factor in multiple cell and animal models [26–30]. We showed that FTY720 improves GI physiology/function and reduces GI aSyn pathology in A53T Tg parkinsonian mice [22]. In CNP-aSyn MSA mice we have assessed FTY720-Mitoxy, our novel neuroprotective FTY720-derivative, [27] that though not orally bioavailable, readily crosses the blood brain barrier [31], stimulates trophic factor expression in neurons and oligodendroglia [32, 33], and is not immunosuppressive by decreasing circulating T cells [34]. Preliminary data confirm the restorative effects of FTY720-Mitoxy on motor, sudomotor, and GI function in Tg CNP-aSyn MSA mice [21].

In this pilot study we evaluated the gut microbiota of human PD and control subjects and parkinsonian A53T aSyn and MSA CNP-aSyn Tg mouse models. We also sought to determine if mice and humans have shared GI microbiota and if potential therapeutic drugs for PD and/or MSA may affect the microbiota of mice, in hopes of producing information that may translate to humans.

## METHODS

### Human subjects

Our human study was ethically performed as approved by the Texas Tech University Health Science Center (TTUHSC) El Paso Institutional Review Board (protocol #E16039). Subjects gave written informed consent to provide samples and health information that were de-identified and HIPAA protected. In 9 subjects with PD and 13 healthy controls, participants were recruited by flyers posted in clinics and hospitals and also by inviting subjects who attended local PD group meetings and educational programs. Inclusion criteria for PD subjects were: 1) between 35 and 95 years of age, 2) neurologist confirmed PD diagnosis. Exclusion criteria for PD subjects were: 1) dementia, 2) major GI disease or cancer, 3) smoking or use of recreational drugs, 4) medical device implanted near the GI tract, 5) an MRI during the time of the study, 6) pregnancy, or 7) use of antibiotics within 2 weeks prior to initiation of the study. Inclusion criteria for control subjects were: 1) between 35 and 95 years of age, 2) No PD or other neurodegenerative disease, 3) no complaints or symptoms of any disease, 4) no signs of constipation or GI disorder. Exclusion criteria for control subjects were the same as for PD patients.

### Animals and drug delivery

Ethical treatment of animals followed AALAC and NIH guidelines on TTUHSC Institutional Animal Care and Use Committee (IACUC) protocol #12001. A53T aSyn wild type (WT) and transgenic (Tg) mice (B6;C3-Tg-Prnp/SNCA*A53T/83Vle/J; Jackson Laboratories, Bar Harbor, ME) were evaluated at 10 mo and 17 mo after receiving 5–12 mo of oral FTY720 (0.5 mg/kg/mouse) prepared in 200 proof EtOH (vehicle) given 2×/week, while control mice received vehicle alone on the same schedule (N = 17). WT and Tg CNP-aSyn MSA mice (B6;C3-Tg-CNP-SNCA-M2Vle) [25] were assessed at 10–11 mo after receiving 2 or 3 mo of FTY720-Mitoxy (1.1 mg/kg/day) prepared in 200 proof EtOH plus Lactated Ringers (vehicle) or vehicle alone, delivered by Alzet pump Model 2006, implanted subcutaneously on the back of the animal without interfering with normal movement of the mice (N = 10).

### Constipation and gut motility

Participants completed Bristol Stool Form Scale (BSFS) diaries for 14 days, reporting fecal consistency (appearance) and frequency (bowel movements/week) in order to diagnose constipation. Gut motility was measured using Sitzmarks pills (Konsyl Pharmaceuticals, Easton, MD, USA) ingested 5 days before abdominal X-rays, performed using established protocols to quantify radio-opaque markers remaining in the gut as described by other [35, 36].

### Feces collection and DNA extraction

Participants collected fecal samples at home using the provided special container filled with stool DNA stabilizer (PSP® Spin Stool DNA PlusKit, Stratec Molecular) preservative. Specimens were received from all participants and transferred to the laboratory where they were stored at – 80°C until processing. Mouse feces were collected in sterile tubes during 1 hour periods in the morning, with feces preserved and stored as described above. Total fecal DNA was extracted using the QIAamp fast DNA stool Mini kit (Cat # 51604, Qiagen Inc, Valencia, CA) according to manufacturer instructions.

### Sequencing and microbiota analysis

Bacterial 16S rRNA sequencing and microbiota analyses were performed based on the protocol of Reinoso et al [36–38]. Briefly, the *α*- and *β*-diversity were calculated using the microbial ecology (QIIME, version 1.8.0) pipeline and the UniFrac methods, respectively, with the variable region V3 and V4 of bacterial 16S rRNA amplified and sequenced using MiSeq (Illumina Inc., San Diego, CA, USA) at the Texas Tech University Genomics Core (Lubbock, TX).

### Statistical analysis

The two-tailed Mann-Whitney U test was used to analyze relative abundance values for the human study. For animal studies, non-parametric data were evaluated using Clustered Regression Analysis. Spearman correlation analysis was performed to determine the significance levels regarding stool consistency and frequency data for the BSFS analysis. In all cases, p values below .05 were considered significant (CI = 95%) and GraphPad Prism software (v 6.07, San Diego, CA) was used to generate study-specific graphs. Analyses were verified by experts in the Biostatistics and Epidemiology Consulting Lab at TTUHSC El Paso.

## RESULTS

Gut motility was not different between control and PD subjects, with 0–4 markers present in the intestine 5 days after Sitzmarks ingestion, a range that is considered normal. However, the mean BSFS score for consistency was 4.0 and for frequency was 8–16.5 bowel movements/week for controls. In contrast, the PD group had a mean BSFS score of 3.0 and frequency of 5–10, which is consistent with constipation. In PD participants, a moderately high negative correlation (Spearman *ρ*= –0.771, *p* = 0.0426) was found between the mean BSFS consistency and frequency, similar to previous findings by others [39]. Almost no correlation (Spearman *ρ*= 0.110, *p* = 0.747) was established between the mean BSFS consistency and frequency of control participants.

**Fig.1:**
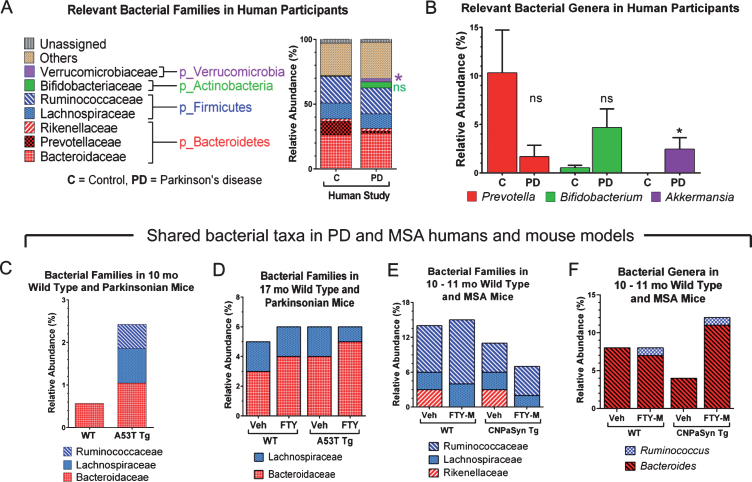
Relative abundance of gut microbial taxa in pilot studies of human PD, and in PD and MSA mouse models and effects of FTY720 (FTY) or FTY720-Mitoxy (FTY-M) on mouse gut microbiota. This figure only shows data relevant to PD and MSA. (A) At the family level Verrucomicrobiaceae (*p < 0.05) is present only in PD subjects and Bifidobacteriaceae (ns) is increased in PD compared to control subjects. (B) The genus *Prevotella* is notoriously reduced in PD, while *Bifidobacterium* and *Akkermansia* are more abundant in PD than in control subjects; ns, not significant, *p < 0.05, Two-tailed Mann-Whitney U Test. Taxa shared by humans and mice in C – F show the mean % relative abundance (RA) only, as raw data were unavailable for statistical analyses. (C) Families Ruminococcaceae and Lachnospiraceae are only present in 10 mo old A53T transgenic (Tg) mice compared to age matched wild type (WT) mice. (D) At 17 mo, family Bacteroidaceae is somewhat increased in WT and Tg mice after FTY, while family Lachnospiraceae is somewhat reduced in response to FTY in Tg mice. (E) In CNP-aSyn MSA mice, family Rikenellaceae is present only in vehicle (Veh) treated WT and Tg CNP-aSyn mice, but absent in FTY-M treated mice; while Ruminococcaceae family tended to increase in WT FTY-M treated mice. (F) The genus *Ruminococcus* is present only in FTY-M treated WT and MSA CNP-aSyn Tg mice while the genus *Bacteroides* increases only after FTY-M in the CNP-aSyn Tg MSA mice. Taxa are color coded according to phyla. Unassigned = non-bacterial fecal components, Other = unidentified bacteria at that particular taxonomic level or bacteria that are not considered relevant to PD or MSA.

Analysis of microbiota revealed that *α*- and *β*-diversity were not significantly different (not shown); though significant differences were seen in % relative abundance (RA) by Mann-Whitney U tests. At the phylum level the mean % RA of combined Firmicutes and Bacteroidetes represented 88% of the reads for controls and 81% for PD (Table 1, column A). Interestingly, the Verrucomicrobiaceae family was only detected in PD subjects but not in controls (Fig. 1A, Table 1, column A). Keshavarzian et al. [40] found a significant increase in the Bacteroidaceae family in their PD subjects compared to controls, though in our study no difference in Bacteroidaceae family was found. Conversely, the Prevotellaceae family was reduced in our PD subjects, though not significantly when compared to control subjects (Fig. 1A and Table 1, column A). At the genus level, putative pro-inflammatory *Akkermansia*, a gram-negative anaerobic bacterium [41], was significantly more abundant in our PD subjects (Mann-Whitney U, *p* = 0.0172) compared to controls, consistent with the results of others [40, 42]. Meanwhile in our pilot study, *Prevotella,* a mucin degrader, was decreased in PD patients as compared to controls although not significantly (*p* = 0.196) (Fig. 1B). A decrease in *Prevotella* is known to be associated with increased gut permeability in association with decreased mucin synthesis [14]. Interestingly, in 17 mo A53T mice, genus *Prevotella* was also increased in parkinsonian Tg animals (Table 1, column C). *Bifidobacterium* is known to be increased in PD patients [14, 40, 42–45] and we have confirmed this finding in our group of PD subjects (*p* = 0.072) and also in 17 mo old vehicle treated parkinsonian A53T Tg mice. Unfortunately, those results did not reach statistically significant levels (Table 1, columns A and C; Fig. 1B, ns). Surprisingly, 10 mo A53T Tg mice had both Proteobacteria and Actinobacteria as their most abundant phyla, contrasting with low % RA of Firmicutes and Bacteroidetes, both of which are normally abundant phyla in humans and mice (Table 1, column B). At 17 mo, parkinsonian A53T Tg mice had a higher % RA of Firmicutes and Bacteroidetes, compared to low reads for these same phyla at 10 mo, indicating a possible age-related microbiota change (Table 1, columns B and C). The Lachnospiraceae family in the Firmicutes was shared between humans, A53T Tg vehicle 10 mo, and both WT and Tg A53T mice at 17 mo (Fig. 1A, C, D and Table 1, columns A–C).

**Table 1:** Relative abundance of relevant taxa present in a pilot study comparing Parkinson’s disease participants with controls and taxa shared with parkinsonian and multiple system atrophy mouse models

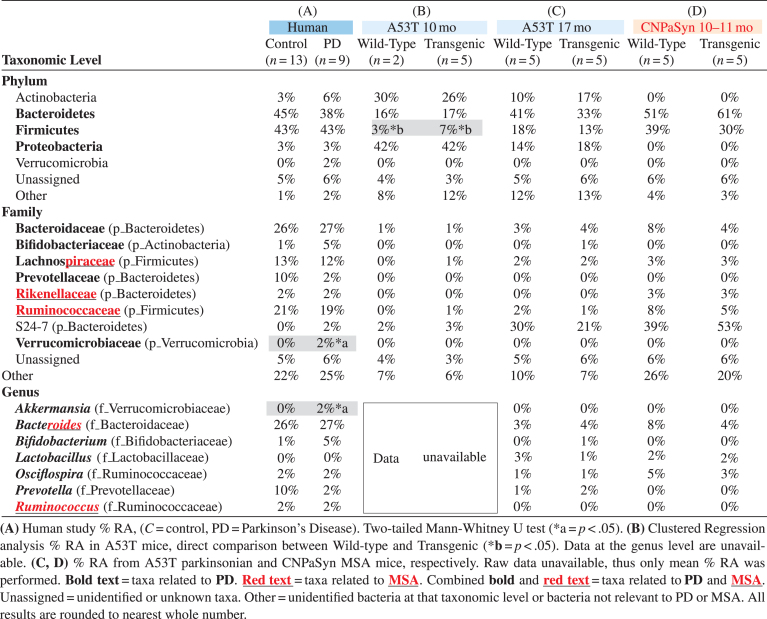

An effect on the families Lachnospiraceae and Bacteroidaceae was observed after treating A53T WT and Tg mice with FTY720 (Fig. 1D). The Lachnospiraceae family is of special interest because it is reportedly reduced in some PD patients [40]. Interestingly, Lachnospiraceae produce short chain fatty acids in the human gut [44] and its depletion in PD subjects may partially explain their related GI inflammation/dysfunction that occurs, as some short chain fatty acids are able to reduce inflammation [16, 44]. The Bacteroidaceae and S24-7 families represent shared taxa present in all subjects and all conditions studied (Fig. 1A, 1C, 1D and Table 1, columns A–D). According to Keshavarzian et al [40], Bacteroidaceae family is significantly more abundant in PD as compared to controls. The relevance of the S24-7 family is still unclear and needs further investigation, although existing links are found between increased S24-7 abundance and high-fat fed diabetes-sensitive-mice [46].

Firmicutes and Bacteroidetes represent the most abundant phyla found in CNP-aSyn MSA mice (Table 1, column D). Lachnospiraceae, Rikenellaceae, and Ruminococcaceae families present in CNP-aSyn mice were also shared with humans (Table 1, columns A and D). The putative anti-inflammatory bacteria families Lachnospiraceae and Ruminococcaceae are reported to be reduced in MSA patients compared to controls [17]. The Ruminococcaceae family was reduced in MSA CNP-aSyn Tg mice compared to WT and when mice were FTY720-Mitoxy-treated, this family showed little or no change in % RA, with a greater change noted for FTY720-Mitoxy treated WT mice (Fig. 1E). Authors, including Engen et al, previously reported significantly higher abundance of the Rikenellaceae family in MSA subjects as compared to controls [17]. Notably, the Rikenellaceae family was not detected after FTY720-Mitoxy treatment, an effect that may also be of benefit for MSA patients (Fig. 1E). *Ruminococcus*, which are short chain fatty acid/butyrate-producing anti-inflammatory bacteria, were present at 1% RA in FTY720-Mitoxy treated mice. *Ruminococcus* is reportedly low in MSA, so administering a drug that increases its abundance might benefit MSA patients (Fig. 1F). *Bacteriodes*, which is more abundant in MSA, was also more abundant in MSA-like vehicle treated CNP-aSyn Tg mice (Fig. 1F, Table 1, column D).

## DISCUSSION

A recent review by Gerhardt and Mohajeri [42] includes data about colonic bacterial composition in PD and other neurodegenerative diseases and reported *β*-diversity that was significantly different between healthy controls and PD in all studies reviewed. In this pilot study *β*-diversity was not found to be statistically significantly different between our PD and control subjects. In that same Gerhardt and Mohajeri review three studies reported that the genus *Bifidobacterium* is significantly increased, and *Prevotella* is significantly reduced in PD as compared to controls. In our pilot study we noted a trend toward an increase in *Bifidobacterium* and a reduction in *Prevotella* but those data did not reach significance. Despite the fact that some of our findings did not replicate data of others, we did reproduce the changes seen in *Akkermansia* as reported by Keshavarsian et al. [40], and were also able to establish a trend toward a decrease in the presence of Prevotellaceae as shown by Unger et al. [43]. A limitation of this pilot study is possibly related to the small number of participants, as we believe this fact could have affected the sensitivity of the results. Other aspects that may have contributed to our findings are differences in DNA extraction methodology, use of medications by our PD participants and/or differences in disease duration in our PD subjects, as this has been observed to affect data obtained by others [42, 44].

In conclusion, human PD gut microbiota changes in *Akkermansia* and Prevotellaceae were reproduced in our study, further supporting their use as biomarkers for early PD diagnosis. Additional work should test this possibility as well as evaluate identified shared microbial markers that correlated with disease in our PD and MSA mouse models. Our results suggest that mice may be useful surrogates for evaluating therapeutic effects on gut function and/or pathology, as we did with FTY720 in parkinsonian A53T Tg mice [22, 26, 28]. Although here we saw little impact of FTY720 on mouse microbiota, desirable microbial effects were noted in response to FTY720-Mitoxy, supporting further evaluation of this anti-synucleinopathy, neurotrophic-factor-enhancing, brain penetrating compound [32, 33].

## CONFLICT OF INTEREST

The corresponding author has filed a patent “Compositions and Methods for the Treatment of Parkinson’s Disease”, US Patent No. 10,391,066 (issued), CA 2888634 (pending), MX 004806 (pending); which does not alter adherence to Journal of Parkinson’s Disease policies.

## References

[1] Aron-Wisnewsky J , Clement K (2016) The gut microbiome, diet, and links to cardiometabolic and chronic disorders. Nat Rev Nephrol 12, 169–181. 26616538 10.1038/nrneph.2015.191

[2] LeBlanc JG , Chain F , Martin R , Bermudez-Humaran LG , Courau S , Langella P (2017) Beneficial effects on host energy metabolism of short-chain fatty acids and vitamins produced by commensal and probiotic bacteria. Microb Cell Fact 16, 79. 28482838 10.1186/s12934-017-0691-z PMC5423028

[3] Serino M , Luche E , Gres S , Baylac A , Berge M , Cenac C , Waget A , Klopp P , Iacovoni J , Klopp C , Mariette J , Bouchez O , Lluch J , Ouarne F , Monsan P , Valet P , Roques C , Amar J , Bouloumie A , Theodorou V , Burcelin R (2012) Metabolic adaptation to a high-fat diet is associated with a change in the gut microbiota. Gut 61, 543–553. 22110050 10.1136/gutjnl-2011-301012 PMC3292714

[4] Belizário JE , Faintuch J , Garay-Malpartida M (2018) Gut microbiome dysbiosis and immunometabolism: New frontiers for treatment of metabolic diseases. Mediators Inflamm 2018, 2037838. 30622429 10.1155/2018/2037838 PMC6304917

[5] Dinan TG , Cryan JF (2017) Gut instincts: Microbiota as a key regulator of brain development, ageing and neurodegeneration. J Physiol 595, 489–503. 27641441 10.1113/JP273106 PMC5233671

[6] Sampson TR , Debelius JW , Thron T , Janssen S , Shastri GG , Ilhan ZE , Challis C , Schretter CE , Rocha S , Gradinaru V , Chesselet MF , Keshavarzian A , Shannon KM , Krajmalnik-Brown R , Wittung-Stafshede P , Knight R , Mazmanian SK (2016) Gut microbiota regulate motor deficits and neuroinflammation in a model of Parkinson’s diseasee. Cell 167, 1469–1480 e1412. 27912057 10.1016/j.cell.2016.11.018 PMC5718049

[7] O’Mahony SM , Clarke G , Borre YE , Dinan TG , Cryan JF (2015) Serotonin, tryptophan metabolism and the brain-gut-microbiome axis. Behav Brain Res 277, 32–48. 25078296 10.1016/j.bbr.2014.07.027

[8] Maier L , Pruteanu M , Kuhn M , Zeller G , Telzerow A , Anderson EE , Brochado AR , Fernandez KC , Dose H , Mori H , Patil KR , Bork P , Typas A (2018) Extensive impact of non-antibiotic drugs on human gut bacteria. Nature 555, 623–628. 29555994 10.1038/nature25979 PMC6108420

[9] Kuang YS , Lu JH , Li SH , Li JH , Yuan MY , He JR , Chen NN , Xiao WQ , Shen SY , Qiu L , Wu YF , Hu CY , Wu YY , Li WD , Chen QZ , Deng HW , Papasian CJ , Xia HM , Qiu X (2017) Connections between the human gut microbiome and gestational diabetes mellitus. Gigascience 6, 1–12. 10.1093/gigascience/gix058 PMC5597849 28873967

[10] Petersen C , Wankhade UD , Bharat D , Wong K , Mueller JE , Chintapalli SV , Piccolo BD , Jalili T , Jia Z , Symons JD , Shankar K , Anandh Babu PV (2019) Dietary supplementation with strawberry induces marked changes in the composition and functional potential of the gut microbiome in diabetic mice. J Nutr Biochem 66, 63–69. 30771735 10.1016/j.jnutbio.2019.01.004 PMC6490960

[11] Halfvarson J , Brislawn CJ , Lamendella R , Vázquez-Baeza Y , Walters WA , Bramer LM , D’Amato M , Bonfiglio F , McDonald D , Gonzalez A , McClure EE , Dunklebarger MF , Knight R , Jansson JK (2017) Dynamics of the human gut microbiome in inflammatory bowel disease. Nat Microbiol 2, 17004. 28191884 10.1038/nmicrobiol.2017.4 PMC5319707

[12] Sanmiguel C , Gupta A , Mayer EA (2015) Gut microbiome and obesity: A plausible explanation for obesity. Curr Obes Rep 4, 250–261. 26029487 10.1007/s13679-015-0152-0 PMC4443745

[13] Cersosimo MG , Benarroch EE (2012) Autonomic involvement in Parkinson’s disease: Pathology, pathophysiology, clinical features and possible peripheral biomarkers. J Neurol Sci 313, 57–63. 22001247 10.1016/j.jns.2011.09.030

[14] Scheperjans F , Aho V , Pereira PA , Koskinen K , Paulin L , Pekkonen E , Haapaniemi E , Kaakkola S , Eerola-Rautio J , Pohja M , Kinnunen E , Murros K , Auvinen P (2015) Gut microbiota are related to Parkinson’s disease and clinical phenotype.350-358. Mov Disord 30. 10.1002/mds.26069 25476529

[15] Mulak A , Bonaz B (2015) Brain-gut-microbiota axis in Parkinson’s disease. World J Gastroenterol 21, 10609–10620. 26457021 10.3748/wjg.v21.i37.10609 PMC4588083

[16] Tan AH , Chong CW , Song SL , Teh CSJ , Yap IKS , Loke MF , Tan YQ , Yong HS , Mahadeva S , Lang AE , Lim SY (2018) Altered gut microbiome and metabolome in patients with multiple system atrophy. Mov Disord 33, 174–176. 10.1002/mds.27203 29083071

[17] Engen PA , Dodiya HB , Naqib A , Forsyth CB , Green SJ , Voigt RM , Kordower JH , Mutlu EA , Shannon KM , Keshavarzian A (2017) The potential role of gut-derived inflammation in multiple system atrophy. J Parkinsons Dis 7, 331–346. 28234259 10.3233/JPD-160991

[18] Scheperjans F , Derkinderen P , Borghammer P (2018) The gut and Parkinson’s disease: Hype or hope? J Parkinsons Dis 8, S31–S39. 30584161 10.3233/JPD-181477 PMC6311363

[19] Giasson BI , Duda JE , Quinn SM , Zhang B , Trojanowski JQ , Lee VM (2002) Neuronal alpha-synucleinopathy with severe movement disorder in mice expressing A53T human alpha-synuclein. Neuron 34, 521–533. 12062037 10.1016/s0896-6273(02)00682-7

[20] Yazawa I , Giasson BI , Sasaki R , Zhang B , Joyce S , Uryu K , Trojanowski JQ , Lee VM (2005) Mouse model of multiple system atrophy alpha-synuclein expression in oligodendrocytes causes glial and neuronal degeneration. Neuron 45, 847–859. 15797547 10.1016/j.neuron.2005.01.032

[21] Vidal-Martinez G , Yang B , Segura-Ulate I , Vargas-Medrano J , Chaparro S , Arterburn JB , Sandoval H , Perez RG (2017) Assessing FTY720-mitoxy, a novel fingolimod-derivative, in mice as a therapy for multiple system atrophy (MSA). *Society for Neuroscience* 2017 Neuroscience Meeting Planner. Online. Washington, DC, Program Number 300.04.

[22] Vidal-Martinez G , Vargas-Medrano J , Gil-Tommee C , Medina D , Garza NT , Yang B , Segura-Ulate I , Dominguez SJ , Perez RG (2016) FTY720/Fingolimod reduces synucleinopathy and improves gut motility in A53T Mice: Contributions of pro-brain-derived neurotrophic factor (Pro-BDNF) and mature BDNF. J Biol Chem 291, 20811–20821. 27528608 10.1074/jbc.M116.744029 PMC5034069

[23] Perez-Pardo P , Dodiya HB , Engen PA , Forsyth CB , Huschens AM , Shaikh M , Voigt RM , Naqib A , Green SJ , Kordower JH , Shannon KM , Garssen J , Kraneveld AD , Keshavarzian A (2019) Role of TLR4 in the gut-brain axis in Parkinson’s disease: A translational study from men to mice. Gut 68, 829–843. 30554160 10.1136/gutjnl-2018-316844

[24] Perez-Pardo P , Dodiya HB , Engen PA , Naqib A , Forsyth CB , Green SJ , Garssen J , Keshavarzian A , Kraneveld AD (2018) Gut bacterial composition in a mouse model of Parkinson’s disease. Benef Microbes 9, 799–814. 30099890 10.3920/BM2017.0202

[25] Brinkmann V , Billich A , Baumruker T , Heining P , Schmouder R , Francis G , Aradhye S , Burtin P (2010) Fingolimod (FTY720): Discovery and development of an oral drug to treat multiple sclerosis. Nat Rev Drug Discov 9, 883–897. 21031003 10.1038/nrd3248

[26] Ren M , Han M , Wei X , Guo Y , Shi H , Zhang X , Perez RG , Lou H (2017) FTY720 attenuates 6-OHDA-associated dopaminergic degeneration in cellular and mouse parkinsonian models. Neurochem Res 42, 686–696. 27943027 10.1007/s11064-016-2125-4

[27] Vargas-Medrano J , Krishnamachari S , Villanueva E , Godfrey WH , Lou H , Chinnasamy R , Arterburn JB , Perez RG (2014) Novel FTY720-based compounds stimulate neurotrophin expression and phosphatase activity in dopaminergic cells. ACS Med Chem Lett 5, 782–786. 25050165 10.1021/ml500128g PMC4094248

[28] Vidal-Martinez G , Najera K , Miranda JD , Gil-Tommee C , Yang B , Vargas-Medrano J , Diaz-Pacheco V , Perez RG (2019) FTY720 improves behavior, increases brain derived neurotrophic factor and reduces *α*-synuclein pathology in parkinsonian GM2+/–mice. Neuroscience 411, 1–10. 31129200 10.1016/j.neuroscience.2019.05.029 PMC6612448

[29] Vidal-Martinez G , Yang B , Vargas-Medrano J , Perez RG (2018) Could alpha-synuclein modulation of insulin and dopamine identify a novel link between Parkinson’s disease and diabetes as well as potential therapies? Front Mol Neurosci 11, 465. 30622456 10.3389/fnmol.2018.00465 PMC6308185

[30] Deogracias R , Yazdani M , Dekkers MP , Guy J , Ionescu MC , Vogt KE , Barde YA (2012) Fingolimod, a sphingosine-1 phosphate receptor modulator, increases BDNF levels and improves symptoms of a mouse model of Rett syndrome. Proc Natl Acad Sci U S A 109, 14230–14235. 22891354 10.1073/pnas.1206093109 PMC3435172

[31] Enoru JO , Yang B , Krishnamachari S , Villanueva E , DeMaio W , Watanyar A , Chinnasamy R , Arterburn JB , Perez RG (2016) Preclinical metabolism, pharmacokinetics and *in vivo* analysis of new blood-brain-barrier penetrant fingolimod analogues: FTY720-C2 and FTY720-Mitoxy. PLoS One 11, e0162162. 27611691 10.1371/journal.pone.0162162 PMC5017749

[32] Vargas-Medrano J , Segura-Ulate I , Yang B , Chinnasamy R , Arterburn JB , Perez RG (2019) FTY720-mitoxy reduces toxicity associated with alpha-synuclein and oxidative stress by increasing trophic factor expression and myelin protein in OLN-93 oligodendroglia cell cultures. Neuropharmacology 158, 107701. 31291595 10.1016/j.neuropharm.2019.107701 PMC6745250

[33] Vargas-Medrano J , Yang B , Garza NT , Segura-Ulate I , Perez RG (2018) Up-regulation of protective neuronal MicroRNAs by FTY720 and novel FTY720-derivatives. Neurosci Lett 690, 178–180. 30359694 10.1016/j.neulet.2018.10.040 PMC7952001

[34] Segura-Ulate I , Belcher TK , Vidal-Martinez G , Vargas-Medrano J , Perez RG (2017) FTY720-derivatives do not induce FTY720-like lymphopenia. J Pharmacol Sci 133, 187–189. 28363412 10.1016/j.jphs.2017.02.006 PMC7959251

[35] Rao S , Camilleri M , L. Hasler W , Maurer AH , P. Parkman H , Saad R , Scott M , Simren M , Soffer E , Szarka L (2011) Evaluation of gastrointestinal transit in clinical practice: Position paper of the American and European Neurogastroenterology and Motility Societies. Neurogastroenterol Motil 23, 8–23. 21138500 10.1111/j.1365-2982.2010.01612.x

[36] Sarosiek I , H Selover K , A Katz L , R Semler J , E Wilding G , Lackner J , D Sitrin M , Kuo B , Chey W , L Hasler W , L Koch K , P Parkman H , Sarosiek J , W McCallum R (2009) The assessment of regional gut transit times in healthy controls and patients with gastroparesis using wireless motility technology. Aliment Pharmacol Ther 31, 313–322. 19814743 10.1111/j.1365-2036.2009.04162.x PMC4444219

[37] Reinoso Webb C , den Bakker H , Koboziev I , Jones-Hall Y , Rao Kottapalli K , Ostanin D , Furr KL , Mu Q , Luo XM , Grisham MB (2018) Differential susceptibility to t cell-induced colitis in mice: Role of the intestinal microbiota. Inflamm Bowel Dis 24, 361–379. 29361089 10.1093/ibd/izx014 PMC6176899

[38] Reinoso Webb C , Koboziev I , Furr KL , Grisham MB (2016) Protective and pro-inflammatory roles of intestinal bacteria. Pathophysiology 23, 67–80. 26947707 10.1016/j.pathophys.2016.02.002 PMC4867289

[39] Vandeputte D , Falony G , Vieira-Silva S , Tito RY , Joossens M , Raes J (2016) Stool consistency is strongly associated with gut microbiota richness and composition, enterotypes and bacterial growth rates. Gut 65, 57–62. 26069274 10.1136/gutjnl-2015-309618 PMC4717365

[40] Keshavarzian A , Green SJ , Engen PA , Voigt RM , Naqib A , Forsyth CB , Mutlu E , Shannon KM (2015) Colonic bacterial composition in Parkinson’s disease. Mov Disord 30, 1351–1360. 26179554 10.1002/mds.26307

[41] Derrien M , Vaughan EE , Plugge CM , de Vos WM (2004) Akkermansia muciniphila gen. nov., sp. nov., a human intestinal mucin-degrading bacterium. Int J Syst Evol Microbiol 54, 1469–1476. 15388697 10.1099/ijs.0.02873-0

[42] Gerhardt S , Mohajeri MH (2018) Changes of colonic bacterial composition in Parkinson’s disease and other neurodegenerative diseases. Nutrients 10, E708. 29857583 10.3390/nu10060708 PMC6024871

[43] Unger MM , Spiegel J , Dillmann KU , Grundmann D , Philippeit H , Burmann J , Fassbender K , Schwiertz A , Schafer KH (2016) Short chain fatty acids and gut microbiota differ between patients with Parkinson’s disease and age-matched controls. Parkinsonism Relat Disord 32, 66–72. 27591074 10.1016/j.parkreldis.2016.08.019

[44] Hill-Burns EM , Debelius JW , Morton JT , Wissemann WT , Lewis MR , Wallen ZD , Peddada SD , Factor SA , Molho E , Zabetian CP , Knight R , Payami H (2017) Parkinson’s disease and Parkinson’s disease medications have distinct signatures of the gut microbiome. Mov Disord 32, 739–749. 28195358 10.1002/mds.26942 PMC5469442

[45] Petrov VA , Saltykova IV , Zhukova IA , Alifirova VM , Zhukova NG , Dorofeeva YB , Tyakht AV , Kovarsky BA , Alekseev DG , Kostryukova ES , Mironova YS , Izhboldina OP , Nikitina MA , Perevozchikova TV , Fait EA , Babenko VV , Vakhitova MT , Govorun VM , Sazonov AE (2017) Analysis of gut microbiota in patients with Parkinson’s disease. Bull Exp Biol Med 162, 734–737. 28429209 10.1007/s10517-017-3700-7

[46] Ormerod KL , Wood DL , Lachner N , Gellatly SL , Daly JN , Parsons JD , Dal’Molin CG , Palfreyman RW , Nielsen LK , Cooper MA , Morrison M , Hansbro PM , Hugenholtz P (2016) Genomic characterization of the uncultured Bacteroidales family S24-7 inhabiting the guts of homeothermic animals. Microbiome 4, 36. 27388460 10.1186/s40168-016-0181-2 PMC4936053

